# Soil microbial communities in the face of changing farming practices: A case study in an agricultural landscape in France

**DOI:** 10.1371/journal.pone.0252216

**Published:** 2021-06-17

**Authors:** Laurie Dunn, Christophe Lang, Nicolas Marilleau, Sébastien Terrat, Luc Biju-Duval, Mélanie Lelièvre, Solène Perrin, Nicolas Chemidlin Prévost-Bouré

**Affiliations:** 1 UMR 1347 Agroécologie, INRAE, AgrosupDijon, Université Bourgogne Franche-Comté, Dijon, France; 2 Institut de Recherche Femto-ST, CNRS, Université Bourgogne Franche-Comté, Besançon, France; 3 IRD, UMI 209 UMMISCO, Sorbonne Université, Bondy, France; 4 UMR 1347 Agroécologie, Plateforme GenoSol, INRAE, Dijon, France; Government College University Faisalabad, PAKISTAN

## Abstract

According to biogeography studies, the abundance and richness of soil microorganisms vary across multiple spatial scales according to soil properties and farming practices. However, soil microorganisms also exhibit poorly understood temporal variations. This study aimed at better understanding how soil microbial communities respond to changes in farming practices at a landscape scale over time. A regular grid of 269 sites was set up across a 1,200 ha farming landscape, and soil samples were characterized for their molecular microbial biomass and bacterial richness at two dates (2011 and 2016). A mapping approach highlighted that spatial microbial patterns were stable over time, while abundance and richness levels were modified. The drivers of these changes were investigated though a PLS-PM (partial least square path-modeling) approach. Soil properties were stable over time, but farming practices changed. Molecular microbial biomass was mainly driven by soil resources, whereas bacterial richness depended on both farming practices and ecological parameters. Previous-crop and management effects and a temporal dependence of the microbial community on the historical farming management were also highlighted.

## 1. Introduction

The sustainable use of soils is of key importance in the current context of climate change and of a growing worldwide population. This goal may be reached by preserving soil biodiversity through agroecological practices like reduced tillage, lower chemical inputs or diversification of plant species in crop rotations [1]. Soil microorganisms play a crucial role within this biodiversity. This is especially true in agro-ecosystems, where their abundance (up to 1 billion cells *per* gram of soil) and diversity (up to 1 million species *per* gram of soil) [2], are highly involved in soil fertility and stability: they support plant productivity, determine nutrient and water cycling, the soil structural stability, and plant health [3]. Therefore, understanding how soil microorganisms face changes in farming practices across space and over time is a challenge within the framework of a sustainable agriculture.

Many biogeography studies have addressed the spatial distribution of soil microbial communities, demonstrating that their abundance and diversity follow non-random and heterogeneous distributions across multiple spatial scales like the global scale [4], the continental scale [5], the national scale [6], the regional scale [7, 8] and the landscape scale [9–11]. Whatever the spatial scale, these heterogeneous distributions are first determined by environmental properties [12]. The soil pH is the main driver of the distribution of the soil microbial biomass, diversity and community composition [13–16]. Nevertheless, the spatial patterns of soil microbial abundance, diversity and community composition are also driven by the silt and clay contents, the soil organic matter content or the C:N ratio [15–19], and also by land use type and heterogeneity [20], and to a lesser extent by climate (annual rainfall or temperature) [21]. In addition, from the regional to the landscape and local scales, the abundance and diversity of soil microbial communities are dependent on land use intensity [19] and farming practices like tillage [11], crop rotation and fertilization [22].

Farming practices affect the abundance and diversity of soil microbial communities over short- and long-term scales. Over short-term scales, soil tillage can modify soil microbial niche characteristics by changing physical conditions, modifying the distribution of organic matter and nutrient contents or soil temperature, and in turn modify microbial communities [11, 23–25]. Furthermore, changes in land use type (forest to meadow/cropland) or in aboveground plant communities (e.g. including *Brassicaceae* in the crop rotation) induce changes in soil microbial abundance and diversity [26–29]. Additionally, single or repeated pesticide applications can select particular microorganisms that may become dominant in the microbial community [30, 31]. Other studies are focused on longer time periods and demonstrate that long-term and continuous application of mineral fertilizers modify the soil microbial community composition by decreasing carbon and nitrogen contents, while possibly increasing microbial biomass [22, 32]. Enzymatic activities and microbial biomass increase with mineral fertilizer amount depending on the growing crop [33–35]. Similarly, increasing aboveground plant diversity increases soil microbial biomass over time [36] and soil warming or drying tends to drastically change soil microbial communities over the long-term [37–40]. Nevertheless, very few studies have addressed combinations of farming practices for their mid- or long-term effects on soil microbial communities. For example, no-tillage combined with a 4-year crop rotation increased soil microbial biomass, while microbial communities were clearly distinct following a gradient ranging from plowing to no-tillage [41]. Similarly, in a 5-year experiment combining soil preparation, fertilization and a plant cover, microbial functional activities and diversity were promoted by no-tillage with a cover crop and no N-based fertilization [42]. However, understanding the variation of soil microbial communities over time still needs more investigations so as to understand the combined effects of the various farming practices experienced by soil microbial communities. This could be achieved by evaluating the impact of farming management on soil microorganisms at the landscape scale–the main scale on which farmers rely to make decisions [9]. Farming management is considered over multi-annual time scale and this especially true when considering system in transition. Fewer studies have explored soil microbial dynamics over short-time scales (day, month or season) [37, 43–47] but fewer have assessed longer time scales. Yet, changes in soil microbial communities over several years can change biogeochemical processes and thus soil functions [48].

The objective of this study was to evaluate how soil microbial communities change at the landscape scale over time depending on variation in soil properties, land use and farming practices. A regular grid of 269 sites was set up across Fénay landscape (Burgundy, France)–a farmed landscape composed of 9 km^2^ of croplands and 3 km^2^ of forests–, and each site was sampled in 2011 and 2016. For each site, soil physico-chemical characteristics and farming practices were collected, soil molecular microbial biomass (SMMB) was measured, and bacterial richness was determined by high-throughput sequencing. Then, the spatial patterns of the soil physico-chemical and microbial characteristics of the two sampling campaigns were compared, together with farming practices. SMMB and bacterial richness were modeled using a partial least square path-modeling (PLS-PM). This method permit to identify main drivers of SMMB and bacterial richness by identify impacts of several latent variables composed of various measured variables. For this study, total impacts (direct and indirect) of land use, soil resources, crop rotation and farming intensity on SMMB and bacterial richness were considered and ranked. Temporal evolutions of microbial communities were also considered. We hypothesized that 1) soil microbial abundance, bacterial richness and farming practices changed over time, but soil parameters did not, 2) soil parameters were the main drivers of soil microbial abundance and bacterial richness, and 3) soil microbial abundance and bacterial richness depended on both present and past farming management.

## 2. Materials and methods

### 2.1. Site context

**Fig 1:**
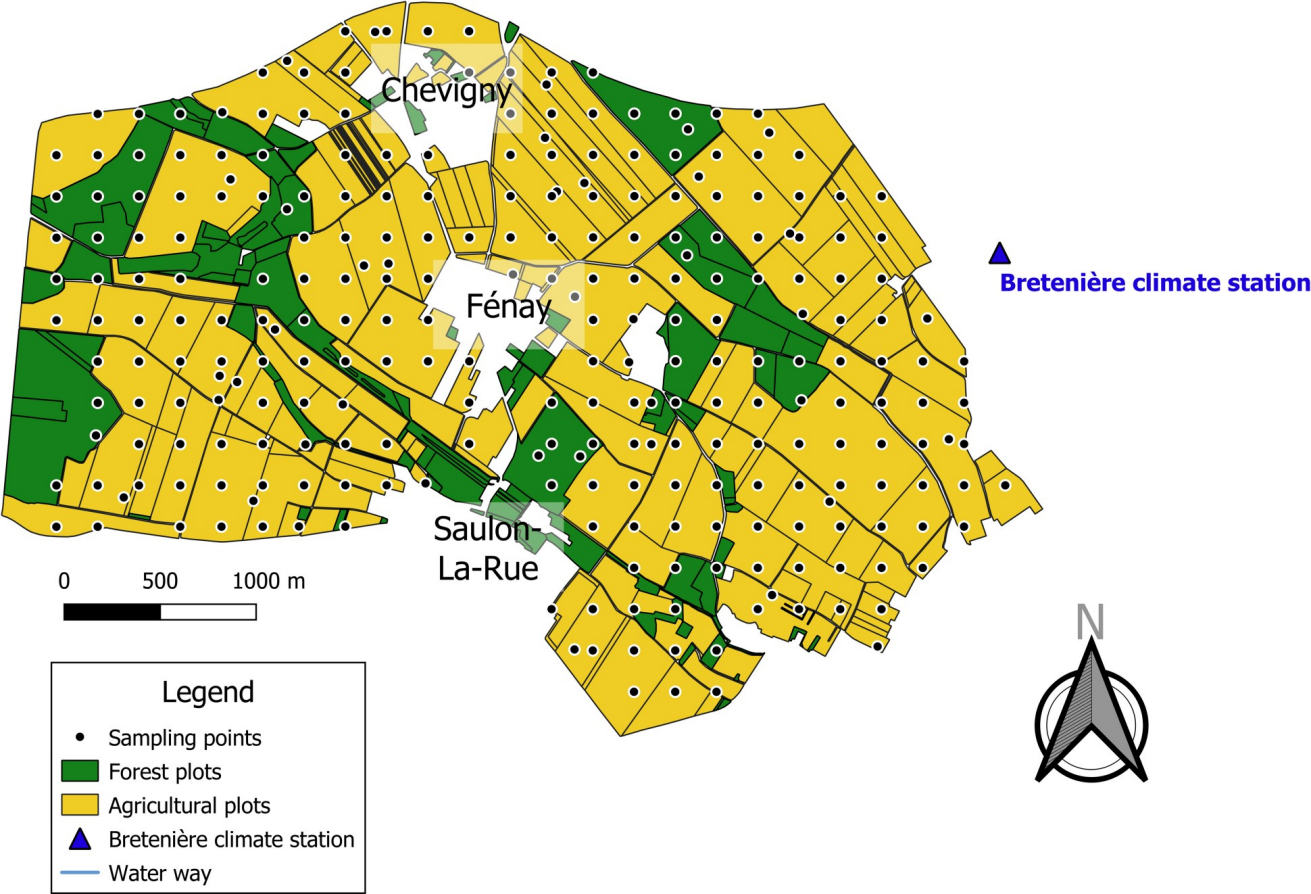
Map of the sampling points across the Fénay landscape, Burgundy, France. Green, forest plots; yellow, farmed plots. Black circles, sampling points; blue triangle, Bretenière climate station. Blue lines are waterways.

The study was conducted on a monitored 12-km^2^ landscape located in Fénay (47°14’37”N, 5°03’36”E) (Burgundy, France). This landscape is composed of approximately 30% of forest plots and 70% of intensive farming plots (Fig 1). Three villages are located in the area: Chevigny up north, Fénay in the center, and Saulon-La-Rue down south. Two rivers flow across the area: La Sans Fond (north west to south) and Le Fossé de Chevigny (north to south east). The climate is continental (average temperature: 10.6°C, average rainfall 768 mm *per* year (https://fr.climate-data.org), and soils are predominantly clayey, clayey-loamy and silty-loamy.

### 2.2. Data collection

#### 2.2.1. Sampling design

The sampling design covered the entire study area. Sampling was carried out in two campaigns (in 2011 and 2016), based on a regular 215 x 215 m grid associated to 30 randomly distributed sites, hence a total of 278 sampling sites. Among these 278 sites, only 269 were retained for analysis. Nine sites were excluded because of incomplete farming management data over the period or inaccessibility or land-use change to urban categories in 2016. Forty-three sites belonged to forest plots, and 226 belonged to farming plots. The minimal distance between two sampling sites was *ca*. 30 m. Soil samples corresponded to a composite of 5 soil cores collected over a 4-m^2^ area (0–20 cm depth). The soil samples were sieved through a 2-mm mesh, lyophilized at -80°C, and stored at -40°C in the soil conservatory of the GenoSol platform, Dijon, France (https://www2.dijon.inrae.fr/plateforme_genosol/) until analysis.

#### 2.2.2. Characterization of soil microbial communities

*2*.*2*.*2*.*1*. *DNA extraction and sequencing*. Soil DNA extraction was performed following the protocol described in [9, 49]. Crude DNA was purified using a Nucleospin Soil PCR purification kit (Macherey Nagel, illkirch, France) and quantified on 1% agarose electrophoresis gels against serial dilutions of calf thymus DNA (Bio-Rad, Hercules, CA, USA). This crude DNA amount corresponded to soil molecular microbial biomass (SMMB) in mg *per* gram of soil. The soil bacterial communities were characterized using two technologies– 454 pyrosequencing (GS FLX Titanium, Roche) in 2011 and MiSeq technology (paired-end reads, 2x300, Illumina) in 2016 –because high-throughput sequencing technologies drastically changed in 2015. Nevertheless, only the sequencing technology changed; the target region remained the bacterial 16S rRNA V3-V4 gene region, and primer pairs and PCR conditions remained the same. A comparative test is provided as (S1 File). Briefly, a 375-bp fragment of the V3-V4 region was amplified using the primer pair F479/R888 (5′-CAG CMG CYG CNG TAA NAC-3′ / 5′-CCG YCA ATT CMT TTR AGT-3′; [50]). Five ng of DNA were amplified in a 25-μL PCR reaction volume in the following conditions: 2 min at 94°C, followed by 35 cycles of 30 s at 94°C, 30 s at 52°C and 1 min at 72°C, and 7 min at 72°C for final elongation. A barcode (a 10-bp multiplex identifier) was added to the amplicons before sequencing [18]. The raw data sets are publicly available in the EBI database system under project accessions no. PRJEB5219 and PRJEB44563 for data in 2011 and 2016 respectively.

*2*.*2*.*2*.*2*. *Bioinformatic analyses*. Bioinformatic analyses were performed with BIOCOM-PIPE [51] and the two field campaigns were analyzed together. First, the 16S raw reads (6,030,013 and 21,072,937 raw reads in 2011 and 2016, respectively) were sorted according to each sample using multiplex identifiers. Low-quality reads were deleted based on their length (< 350 bp), their number of ambiguities and their primer sequence(s); this was followed by rigorous dereplication using a PERL script (i.e. clustering of strictly identical sequences). Dereplicated reads were aligned using Infernal alignment [52] and then clustered at 95% similarity. All single-singletons (reads detected only once and not clustered) were checked to eliminate PCR chimeras and large sequencing errors produced by the PCR and the sequencing, based on the quality of their taxonomic assignments. More precisely, each single-singleton was compared with a dedicated reference database from the SILVA curated database (version R114) using similarity approaches (USEARCH), with sequences longer than 500 nucleotides, and kept only if their identity was higher than the defined threshold. Finally, the number of high-quality reads for each sample was normalized (10,000 high-quality reads for each sample) by random selection to compare the datasets efficiently and avoid biased community comparisons. Then, the ReClustOR algorithm [53] was used to improve sequence assignment to each OTU (Operational Taxonomic Unit, 95% similarity level). Based on the resulting contingency table, bacterial richness was determined, and represented the number of OTUs at the end of the analysis.

#### 2.2.3. Soil physicochemical characteristics

Physicochemical analyses (pH NF ISO 10 390, organic carbon (SOC, g.kg^-1^) NF ISO 10 694, total nitrogen (TN, g.kg^-1^) NF ISO 13 878, clay, silt and sand percentages NF ISO 11277) were performed by the Laboratoire d’analyse des sols d’Arras, Arras, France (https://www6.hautsdefrance.inrae.fr/las). Organic matter (OM) was calculated from SOC multiplied by 1.72. All these analyses were carried out for the two 2011 and 2016 sampling campaigns. Soil water availability was determined based on the cumulative days of water stress *per* year for each sampling site. Daily climate data (temperature and rainfall) were retrieved from the Bretenière climate station, Agroclim, INRAe (Fig 1) for the 2011–2016 period. These measurements were assumed homogeneous for the whole area and used to estimate daily rainfall (RR) and daily potential evapotranspiration using Penman formula (ETPp). First, the available maximum water-holding capacity (WHC) was calculated based on soil granulometry and depth (https://bourgogne.websol.fr), soil textures were classified based on [54], and the WHC *per* soil-depth (cm) was calculated based on [55]. Crop evapotranspiration *per* 10-day period for each plant growth stage was estimated based on their crop coefficient (Aquastat, Food and Agriculture Organization) according to Eq (1):

$$ETM=K_{c}*{ETP}_{p}$$ Eq (1)

where ETM is the maximum crop evapotranspiration, Kc the crop coefficient, and ETPp Penman evapotranspiration in mm.day^-1^. Then, a soil water balance model was used to estimate the cumulative days of water stress [56]. WHC was divided in two compartments: WHC1 (0–20 cm depth) and WHC2 (from 20 cm depth to the bottom of the soil profile). Second, full storage was initialized at maximum capacity for each water storage. Then, each day, storage fillings were calculated considering 1) real evapotranspiration and 2) soil moisture replenishment by rainfall. In this case, soil samples were taken at 0–20 cm depth. Therefore, each day when WHC1 = 0 corresponded to a day of water stress for soil microorganisms. We calculated the cumulative days of water stress (water stress, d.y^-1^) for each year and each sampling point.

#### 2.2.4. Comparison of farming practices

Farming practices were collected from 2004 to 2016. Each year, farmers recorded their farming operations by date: crop type, applications of pesticides or fertilizers (type and quantity), plot area, etc. To compare the sampling campaigns, information was aggregated throughout a farming year from sowing to harvesting (Sept. 2010 to Sept. 2011 for the 2011 campaign, and Sept. 2015 to Sept. 2016 for the 2016 campaign). This aggregation was performed to characterize crop rotations and farming intensity.

Crop rotation was characterized by 6 indicators: 1) the crop type present on the plot (leaving forests aside), i.e., winter crops, spring crops, summer crops, *Brassicaceae* crops, and others (fallow or perennial crops, i.e. blackcurrant, miscanthus); then, for each crop type, its frequency within a 4-year window ending the year of sampling was calculated, leading to 4 indicators: 2) Freq_winter, 3) Freq_spring, 4) Freq_summer, 5) Freq_brassi. Finally, the number of species in the rotation was also estimated on an 8-year basis (nbr_species; 6)).

Farming intensity was characterized by 11 indicators: 1) soil preparation categories were defined the year of sampling as no tillage, intermediate tillage (less than 25 cm depth), decompacting (more than 25 cm depth, no plowing), and plowing. Tillage and plowing frequencies were calculated over a 4-year window ending the year of sampling, leading to 2 indicators: 2) Freq_tillage and 3) Freq_plowing. Based on the applied dose *per* ha relatively to the recommended dose *per* ha, treatment frequency indices were calculated for herbicide (TFI_herbicide, 4), fungicide (TFI_fungicide, 5) and total pesticide (TFI_total, 6) treatments, including anti-slug and insecticide treatments. The use of fertilizers was characterized by the respective total amounts of nitrogen (N, 7), phosphate (P, 8), potassium (K, 9), magnesium (Mg, 10) or sulfur (S, 11) *per* year and *per* ha (kg.ha^-1^.y^-1^).

### 2.3. Statistical analyses

**Fig 2:**
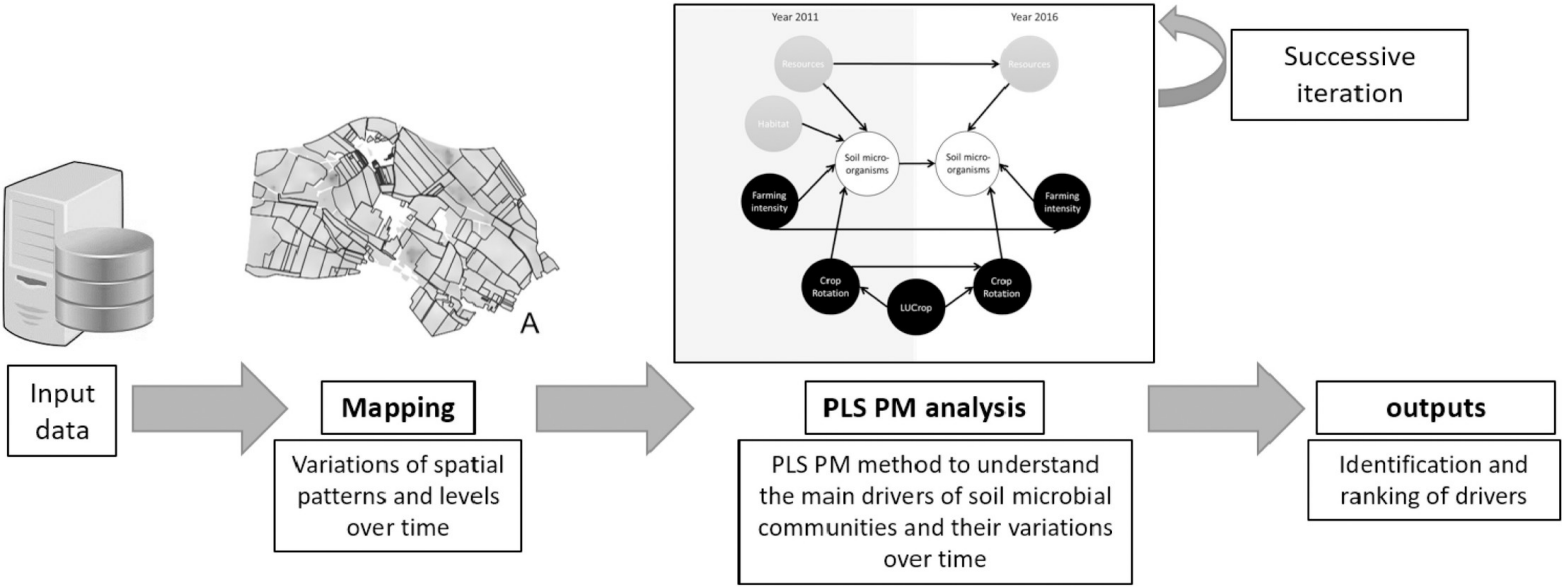
Analytical workflow. Two main analyses were led: 1) a mapping step identified the variations of the spatial patterns of our variables and potential variations of the levels; 2) a second step based on a PLS PM method identified the main drivers of soil microbial communities and their evolution over time based on the different variables. This part was led with an iterative method where measurable variables, architecture and the significance of interactions were evaluated.

Two main statistical analyses were performed. They are summarized in Fig 2. Technical aspects are described in the following sections.

#### 2.3.1. Interpolated mapping

Most of the variables mentioned above were measured at point sampling. An interpolated mapping approach was used to characterize the spatial patterns of these variables at the landscape scale, following the methodology described in [57]. Most of our data did not follow a normal distribution, so a non-parametric rank order transformation was used before considering spatial correlations and calculating the empirical variogram. Then, a model was fitted to the empirical variogram by weighted non-linear least squares, and validated using a leave-one-out cross-validation approach using the mean square standardized error (MSPE) and the median square standardized error (MedianSPE). To validate the spatial model, errors had to follow a χ^2^ distribution with a mean (Θmean) of 1 and a median (Θmed) of 0.455. Based on these criteria, the best model for the soil and microorganism variables was the Matérn model. Ordinary kriging was estimated in the standardized rank space, and then kriging estimates were back-transformed and plotted [58]. Finally, mean values were compared using a Wilcoxon paired rank test. The GeoR [59] and gstat [60] packages were used in R software [61] for variogram analyses and kriging estimates.

#### 2.3.2. Partial least squares path modeling

Partial least squares path modeling (PLS PM) was used to identify complex multivariate relationships between soil and climate parameters, crop rotations, farming intensity and soil microbial communities. This method is commonly used in social science and is suitable for landscape ecology [62]. It consists in building latent variables (LVs) composed of one or more measured variables (MVs). LVs can interact with each other [63] and define a more or less complex architecture of the model. This method is similar to the structural equation modelling but use partial least square instead of maximum likelihood as a criterion of assessment. It permits to obtain robust explanatory model [64]. It does not require any assumption about variables or error distribution. In this study, we wanted to understand relationships between SMMB (or bacterial richness) with environmental and anthropogenic variables in an agricultural system. As PLS PM is very useful in establishing causal relationships on natural systems [65] and does not require strong assumption, we found that this method was suitable for this study. However, it should be noted that this method does not allow loop in the system and is still an approximate method like correlative analysis. The model and its results were evaluated as follows: 1) validation of the MV content of each LV; 2) validation of the architecture of the model; 3) bootstrap validation of the path coefficients. Separate PLS PM models were set up to understand changes in soil molecular microbial biomass (SMMB) or soil bacterial richness from 2011 to 2016, but involved similar sets of latent variables. Selection of LVs and MVs was based on the unidimensionality criterion for LVs (Cronbach’s alpha and Dillon-Goldstein’s rho higher than 0.7) and on MV loadings λ (correlation between a MV and its LV), weight and cross-loadings (correlation between a MV and all the different LVs) which had to be positive and higher with their related LV than with other LVs. Any LV and MV that did not respect either the unidimensionality criterion or the loading criteria was excluded from the analysis for both years. The retained latent variables were the following ones: soil LVs, including ‘resources’ (SOC, TN, water stress) and ‘habitat’ (clay and silt contents); farming practice LVs, including ‘farming intensity’ (Freq_plowing, TFI Fungicide, TFI total, P, K and S), ‘crop rotation’ (winter crop category, Nbr_species and Freq_winter), and land use (land use) LVs. LVs belonging to soil and farming practices, except “habitat”, were replicated in 2011 and 2016 to allow for temporal dependencies. Then, various model architectures were tested from simple to complex ones (direct and indirect links, previous effects), as suggested by [66]. The congruent architecture was selected based on the coefficient of determination (R^2^) of the explained LV and on the goodness of fit (GOF). The architecture was considered correct when the GOF was greater than 0.4 and very good when the GOF was greater than 0.7. The models were also retained based on minimal prediction errors, and path coefficients were evaluated based on a bootstrap resampling method (n = 10,000) to validate the overall methodology based on the confidence interval and a t-test. These analyses were carried out using the plspm package [67] in R software.

## 3. Results

### 3.1. Changes in soil properties and farming practices

#### 3.1.1. Soil properties

**Fig 3:**
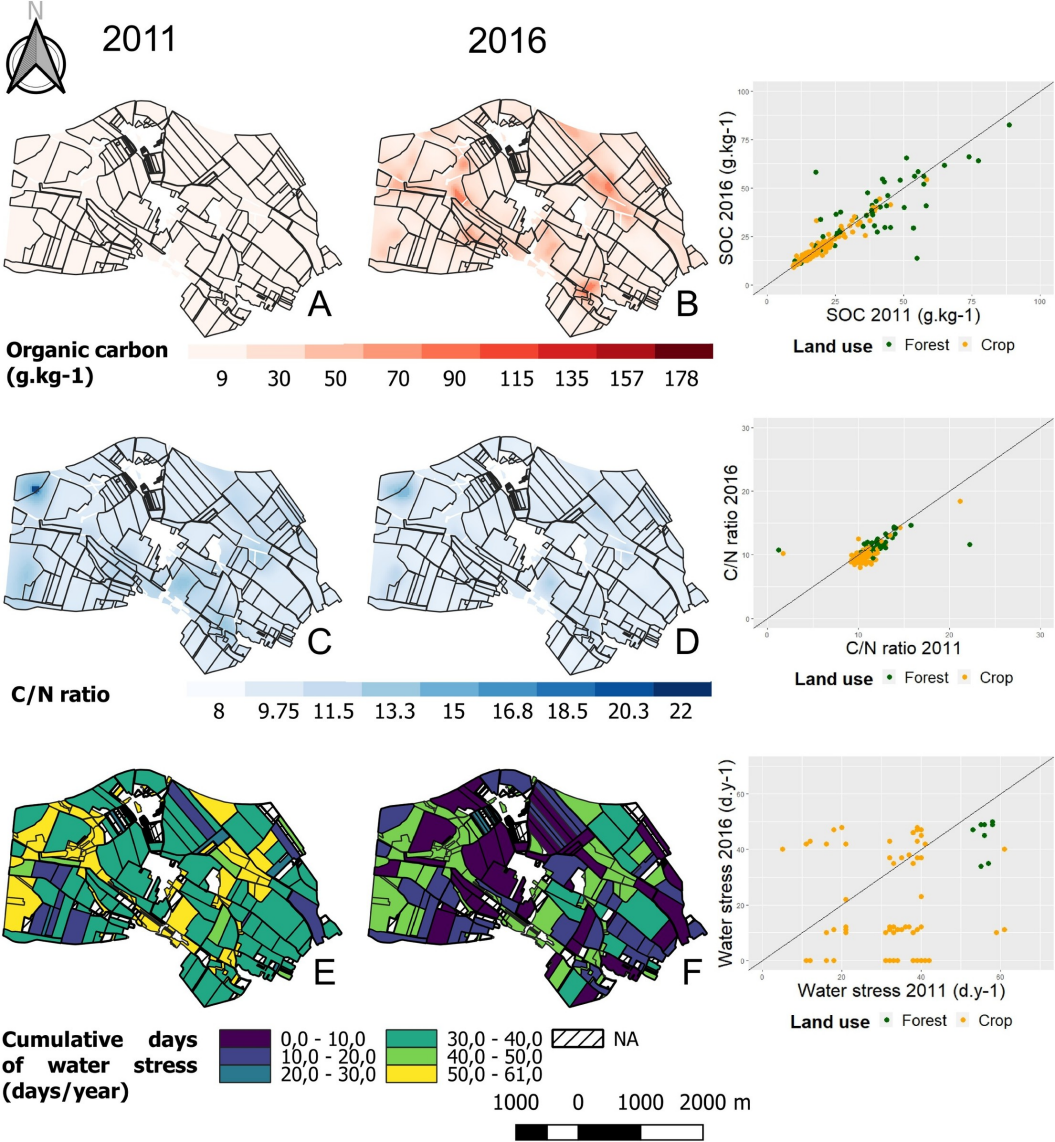
Interpolated mapping of the soil variables organic carbon (A, B), C:N ratio (C, D) and cumulative days of water stress (E, F) for the 2011 sampling campaign (A, C, E) and the 2016 campaign (B, D, F). The Matérn model was used to fit the experimental variogram. The ranges of the models were A: 144.33 m; B: 126.70 m; C: 126.24 m; D: 114.63 m. The estimated water stress in the topsoil is represented at the plot scale. Scatter plots on the right of the figure correspond to the variation of the parameter in 2016 depending on the same parameter in 2011. Green dots, forest plots; orange dots, farmed plots. Plot lines, equation y = x.

Matérn models were used to interpolate soil parameters through a kriging approach. For each model, Θ_mean_ was systematically close to 1 (0.98 < Θ_mean_ < 1.02) and Θ_med_ close to 0.455 (0.34 < Θ_med_ < 0.41). A visual comparison of the maps (Fig 3) highlighted that the spatial patterns of the soil parameters remained unchanged between the two sampling campaigns, as confirmed by the ranges of the respective variograms. Table 1 summarizes the comparison of the two campaigns. At the landscape scale, SOC did not change between 2011 and 2016 (*P* > 0.05) whereas the C:N ratio slightly increased (*P* < 0.001). This difference was related to significant variations in TN (*P* < 0.001) despite very similar mean values: TN decreased from -0.1 to -2 g.kg^-1^ in 209 sites, and increased from 0.1 g.kg^-1^ to 9 g.kg^-1^ in 51 sites. The soil pH slightly decreased over time. The average number of cumulative days of water stress was higher in 2016 than in 2011 (*P* < 0.001).

**Table 1:** Summary of the statistics of microbial variables, soil parameters and farming practices.

Group of variables	Variable	Scale	Mean ± SE in 2011	mean ± SE in 2016	Wilcoxon paired rank test (temporal variation)
**Soil microbial characteristics**	Biomass (mg.g^-1^)	Landscape	64.83 ± 3.42	63.60 ± 2.44	**NS**
		Forest	157.10 ± 12.50	124.37 ± 7.59	**
		Croplands	47.26 ± 1.57	52.03 ± 1.63	.
	Richness (number of OTUs)	Landscape	1936 ± 10.28	1914 ± 11.16	.
		Forest	1773 ± 30.94	1898 ± 28.49	***
		Croplands	1967 ± 9.44	1917 ± 12.15	***
**Soil and climate parameters**	Water stress (d.y^-1^)	Landscape	37.16 ± 0.72	19.71 ± 1.15	***
		Forest	55.35 ± 0.24	48.65 ± 0.12	***
		Croplands	33.7 ± 0.62	14.20 ± 1.02	***
	SOC (g.kg^-1^)	Landscape	21.39 ± 0.78	21.15 ± 0.75	**NS**
		Forest	41.11 ± 2.63	40.22 ± 2.46	**NS**
		Croplands	17.64 ± 0.48	17.52 ± 0.46	**NS**
	TN (g.kg^-1^)	Landscape	2.03 ± 0.08	2.07 ± 0.06	***
		Forest	3.77 ± 0.36	3.51 ± 0.23	**NS**
		Croplands	1.71 ± 0.05	1.79 ± 0.04	***
	C:N	Landscape	10.68 ± 0.10	10.07 ± 0.07	***
		Forest	12.04 ± 0.39	11.69 ± 0.18	*
		Croplands	10.42 ± 0.08	9.76 ± 0.06	***
	pH	Landscape	7.69 ± 0.04	7.63 ± 0.04	***
		Forest	7.12 ± 0.16	7.14 ± 0.15	**NS**
		Croplands	7.80 ± 0.04	7.72 ± 0.03	***
**Habitat**	Clay (%)	Landscape	33.3 ± 0.58		
		Forest	32.60 ± 1.93		
		Croplands	33.44 ± 0.59		
	Silt (%)	Landscape	55.88 ± 0.59		
		Forest	58.45 ± 1.86		
		Croplands	57.77 ± 0.60		
	Sand (%)	Landscape	8.82 ± 0.30		
		Forest	8.95 ± 0.85		
		Croplands	8.79 ± 0.31		
**Crop rotation**	Crop type	Croplands	Winter crop (138). Brassicaceae (45). Summer crop (20). Spring crop (16). Other (7)	Winter crop (141). Brassicaceae (54). Summer crop (18). Other (10). Spring crop (3)	
	Nbr_species	Croplands	4.46 ± 0.06	4.25 ± 0.08	**
	Freq_brassi	Croplands	0.24 ± 0.01	0.26 ± 0.01	**NS**
	Freq_winter	Croplands	0.53 ± 0.01	0.56 ± 0.01	*
	Freq_summer	Croplands	0.08 ± 0.01	0.08 ± 0.01	**NS**
	Freq_spring	Croplands	0.12 ± 0.01	0.07 ± 0.01	***
**Farming intensity**	Soil preparation categories	Croplands	Plowing (108). Intermediate tillage (81). Decompacting (31). No-tillage (5)	Intermediate tillage (69). Decompacting (62). Plowing (54). No-tillage (41)	
	Freq_plowing	Croplands	0.96 ± 0.02	0.41 ± 0.02	***
	Freq_tillage	Croplands	0.58 ± 0.01	0.91 ±0.01	***
	TFI_herbicide	Croplands	1.87 ± 0.06	2.33 ± 0.09	***
	TFI_fungicide	Croplands	1.17 ± 0.05	1.86 ± 0.10	***
	TFI_total	Croplands	4.44 ± 0.013	5.22 ± 0.15	***
	N (kg.ha^-1^)	Croplands	137.20 ± 3.88	157.80 ± 4.07	***
	P (kg.ha^-1^)	Croplands	51.63 ± 1.63	42.68 ± 2.02	***
	K (kg.ha^-1^)	Croplands	35.92 ± 1.60	24.66 ± 1.25	***
	Mg (kg.ha^-1^)	Croplands	6.81 ± 0.44	14.25 ± 0.78	***
	S (kg.ha^-1^)	Croplands	33.48 ± 1.26	52.25 ± 1.29	***

The different mean values and their standard errors were calculated at the landscape, forest and cropland scales. A Wilcoxon paired rank test was run to determine whether the variations between 2011 and 2016 were significant.

In 2011 and in 2016, forest soils had higher pH, SOC and TN, and were dryer than cropland soils (*P* < 0.001). Temporal variations were also observed between 2011 and 2016. They were stronger in croplands than in forests. The C:N ratio and the pH increased while TN and water stress decreased over time in croplands, whereas only water stress and the C:N ratio significantly decreased over time in forests.

#### 3.1.2. Farming practices

**Fig 4:**
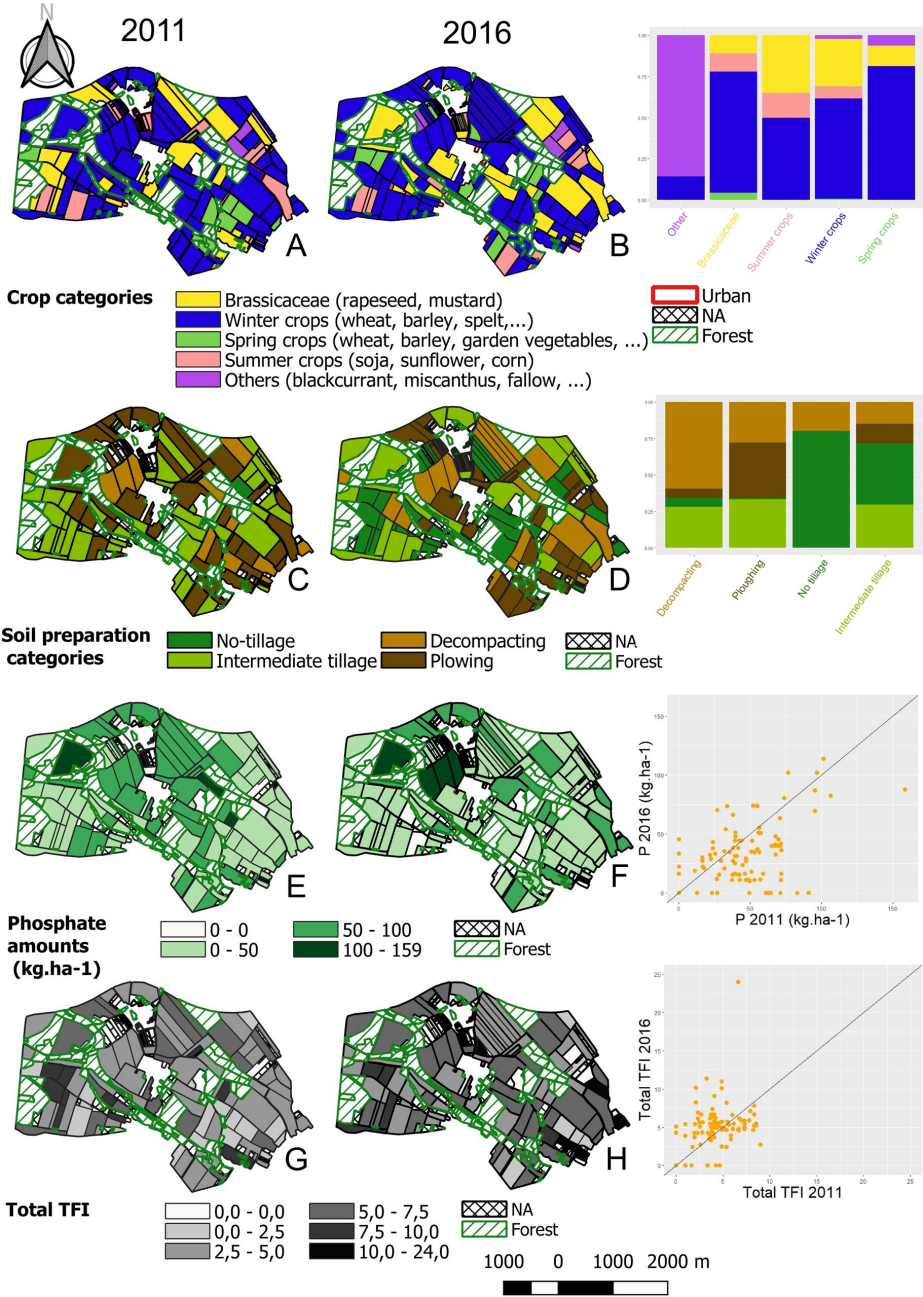
Mapping of the farming practices according to crop categories (A, B), soil preparation categories (C, D), phosphate amounts (E, F) and total TFI (G, H) for the 2011 sampling campaign (A, C, E, G) and the 2016 campaign (B, D, F, H). The legend is the same for both sampling campaigns and is indicated under each pair of maps. Hashed black, plots with missing data (NA); hashed green, forest plots. A comparison between the 2011 and 2016 campaigns is given on the right of the figure, 1) by barplots for crop categories and soil preparation categories, with the proportion of each category in 2016 depending of the category in 2011, and 2) by scatter plots for phosphate amounts and total TFI with the 2016 values as a function of the 2011 values. Only farmed plots (orange dots) are represented. Plot lines, equation y = x.

Farming practices were studied according to crop rotation components (crop categories, crop proportions and frequencies) and farming intensity which integrates soil preparation (categories and frequencies) together with pesticide use as measured by the TFI (total, herbicides and fungicides) and by fertilizer use (N, P, K, Mg, S). The results are mapped in Fig 4. A visual comparison of the maps suggests that farming practices changed between 2011 and 2016. This was confirmed by the statistical comparison of the farming practices between the two sampling campaigns (Table 1).

Considering crop rotation components, the proportion of winter crops was higher in 2011 than in 2016 (61.45% *vs*. 55.84%, respectively). Consequently, the proportion of spring crops was lower in 2011 than in 2016 (6.87% in 2011 *vs*. 8.28% in 2016). Nevertheless, the spring crop frequency (Freq_spring) in the rotations significantly decreased between 2011 and 2016 (*P* < 0.001), while the winter crop frequency (Freq_winter) increased (*P <* 0.05). The number of species in the rotation (Nbr_species) was slightly but significantly lower in 2016 than in 2011 (*P* < 0.01). Fallow fields also increased in 2016 (+ 1.5%) as compared to 2011.

Regarding farming intensity, tillage and plowing decreased at the landscape scale (Table 1) together with Freq_tillage and Freq_plowing in 2016 as compared to 2011 (*P* < 0.001). Pesticide use, i.e. TFI_total, TFI_Herbicide, TFI_Fungicide, significantly increased between 2011 and 2016 (*P* < 0.001). The use of fertilizers was higher in 2016 than in 2011 for N, Mg and S inputs, but lower for P and K inputs (*P* < 0.001).

### 3.2. Soil molecular microbial biomass changes

#### 3.2.1. Spatial patterns and temporal variations

**Fig 5:**
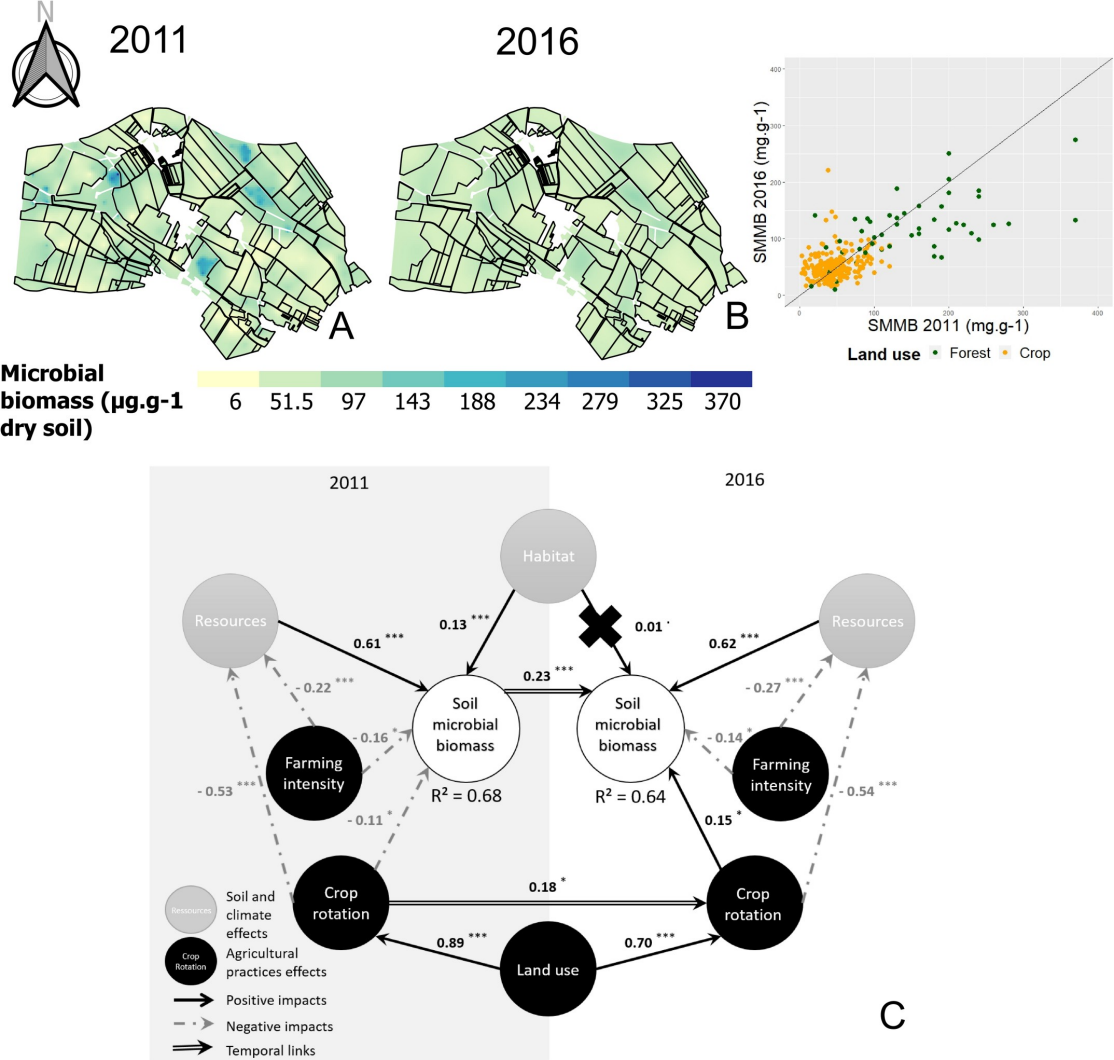
Interpolated mapping of the soil microbial biomass in 2011 (A) and 2016 (B), and complete path model (C). In the interpolated maps, the color scale is the same for the two sampling campaigns and indicates the extrapolated values. The ranges of the models were 199.98 m (A) and 81.69 m (B). The scatter plot on the right of the maps corresponds to the variation of each parameter in 2016 depending on the same parameter in 2011. Green dots, forest plots; orange dots, farmed plots. Line, equation y = x. In the complete path model for biomass analysis (C), circles represent the latent variables (LVs), with anthropogenic LVs in black and ecological LVs in gray. Path coefficients were computed from regressions and allowed us to estimate the strength and direction of relations between LVs. Black arrow, positive impact; gray arrow, negative impact; double arrow, temporal impact. Significant impacts were evaluated based on a t-test: *P* < 0.1; *: *P* < 0.05; **: *P* < 0.01; ***: *P* < 0.001). Crosses indicate inaccurate path coefficients according to a bootstrap validation.

The Matérn model was used to interpolate SMMB through a kriging approach. Θmean was equal to 1.001, and Θmed to 0.447. A visual comparison of SMMB maps suggests that spatial patterns slightly changed between 2011 and 2016, as confirmed by the ranges of the respective variograms (Fig 5), but this variation was not significant at the landscape scale (*P* > 0.05). However, trends were detected based on land use type (forest or cropland). SMMB was significantly higher in forests than in croplands both in 2011 and 2016. Temporal variations were more pronounced in forests than in croplands: they decreased in forests (-20.83%, *P* < 0.01) but increased in croplands (+9.17%, *P* < 0.1).

#### 3.2.2. Soil molecular microbial biomass model

The final architecture of the PLS-PM model for SMMB had a GOF of 0.66 and r^2^ values for soil microbial biomass of 0.67 and 0.65 in 2011 and 2016, respectively (Fig 5). The bootstrap validation suggested that all the different relations between LVs were accurate except the effect of habitat on SMMB in 2016. Based on the absolute value of their total effect (S1 Table), LVs were ranked for their influence on soil molecular microbial biomass. In 2011, the ranking was as follows: soil resources > crop rotation > land use > farming intensity > habitat. In 2016, the ranking changed to soil resources > farming intensity > land use > previous SMMB in 2011 > crop rotation> indirect effects of soil resources, crop rotation and farming intensity in 2011.

Soil resources and habitat had direct positive effects, whereas farming intensity and crop rotation had direct negative effects, except crop rotation in 2016. Soil resources and habitat did not influence SMMB indirectly, whereas farming intensity, crop rotation and land use did. These indirect effects were systematically negative and mediated by soil resources. In the case of land use and crop rotation in 2011, their indirect effects on SMMB in 2016 were mediated by crop rotation in 2016. Similarly, SMMB in 2011 had a strong effect on SMMB in 2016 (β = 0.23).

### 3.3. Soil bacterial richness variations

#### 3.3.1. Spatial patterns and temporal variations

**Fig 6:**
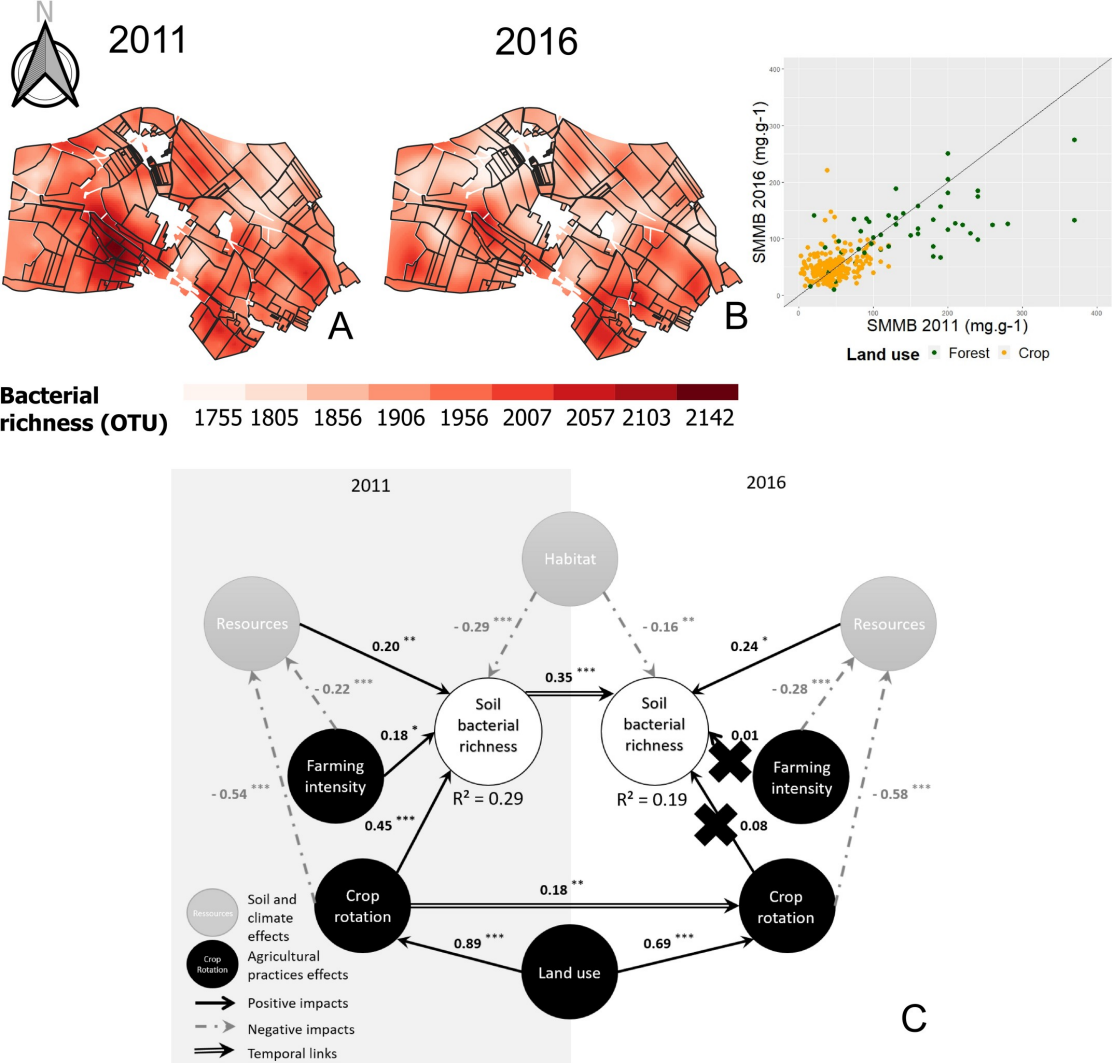
Interpolated mapping of soil bacterial richness in 2011 (A) and 2016 (B), and complete path model (C). In the interpolated maps, the color scale is the same for the two sampling campaigns and indicates the extrapolated values. The ranges of the models were 349.00 m (A) and 101.35 m (B). The scatter plot on the right of the maps corresponds to the variation of the parameter in 2016 depending on the same parameter in 2011. Green dots, forest plots; orange dots, farmed plots. Line, equation y = x. Concerning the complete path model for biomass analysis (C), circles correspond to the latent variables (LVs), with anthropogenic LVs in black and ecological LVs in gray. Path coefficients were computed from regressions and allowed us to estimate the strength and direction of relations between LVs. Black arrow, positive impact; gray arrow, negative impact; double arrow, temporal impact. Significant impacts were evaluated based on a t-test: *P* < 0.1; *: *P* < 0.05; **: *P* < 0.01; ***: *P* < 0.001). Crosses indicate inaccurate path coefficients according to a bootstrap validation.

The Matérn model was used to interpolate soil bacterial richness through a kriging approach. Θ_mean_ was equal to 0.998, and Θ_med_ to 0.509. A visual comparison of the 2011 and 2016 maps suggests that spatial patterns slightly changed, as confirmed by the ranges of the respective variograms (Fig 6). At the landscape scale, richness values slightly changed between 2011 and 2016, but differences were not significant (*P* > 0.05). Nevertheless, significant temporal variations were observed based on land use type. Bacterial richness increased in forest plots (+6.59%, *P* < 0.001) and slightly decreased in farmed plots (-2.54%, *P* < 0.001). These variations were not attributable to the change in the sequencing method between 2011 and 2016 because Illumina sequencing is close to 454 pyrosequencing, and Illumina systematically underestimates soil bacterial richness (mean of 5.94%, S1 File). Bacterial richness was significantly lower in forests than in croplands only in 2011 (*P* < 0.001), but no significant variation was found between forests and croplands in 2016 (Wilcoxon rank test, *P* > 0.1).

#### 3.3.2. Soil bacterial richness model

The final architecture of the PLS-PM model for soil bacterial richness had a GOF of 0.59 and r^2^ values for soil bacterial richness of 0.29 and 0.19 in 2011 and 2016, respectively (Fig 6). Bootstrap validation suggested that direct effects of anthropogenic activities (crop rotation and farming intensity) on soil bacterial richness were not accurate in 2016. Based on the absolute value of total effects (S1 Table), LVs were ranked for their influence on soil bacterial richness. In 2011, soil bacterial richness was driven by crop rotation > land use > habitat > resources > farming intensity. In 2016, the ranking changed to previous soil bacterial richness in 2011 > habitat > resources > indirect effects of crop rotation and resources in 2011 > land use > farming intensity > crop rotation > indirect effect of farming intensity in 2011.

Soil resources and habitat only had direct but opposite effects on soil bacterial richness. Soil resources had a positive effect, but habitat had a negative effect. Crop rotation and farming intensity both had direct positive effects and indirect negative effects on soil bacterial richness. These indirect negative effects were lower than the direct effects, and mediated by soil resources themselves negatively affected by crop rotation and farming intensity. The indirect positive effect of land use and crop rotation in 2011 on soil bacterial richness in 2016 was mediated by the relationship between crop rotation and soil resources in 2016. Soil bacterial richness in 2016 was largely dependent on soil bacterial richness in 2011.

## 4. Discussion

### 4.1. Soil molecular microbial biomass

SMMB was significantly structured across space and heterogeneously distributed both in 2011 and 2016, in agreement with other studies at larger scales [6]. Forests had systematically higher SMMB and lower bacterial richness than croplands, in agreement with the literature [68, 69]. The model explaining SMMB explored the direct and indirect effects of soil resources and habitat (i.e. ecological parameters), of farming practices through farming intensity, land use and crop rotation, but also the previous effects of farming practices and soil microbial characteristics. Soil resources (SOC, TN and water stress) and soil habitat (clay and silt contents) had significant direct positive effects on SMMB, in agreement with other studies [12, 13, 70–73]. This positive effect of soil properties may be related to their slight increase/decrease, as SOC or TN variations over long-term scales may lead to drastic changes in soil microbial communities [74, 75]. Considering water stress, fewer days of water stress were observed in 2016 than in 2011, while SMMB tended to decrease between 2011 and 2016. Therefore, the present model suggests that an increase in the number of days of water stress would increase SMMB. This is quite discussed in the literature, since positive or negative effects of drought have been observed [21, 76]. The positive effect observed in the present study could result from short but frequent water stress periods that reduce the impact of this disturbance.

Farming intensity had a strong negative effect on SMMB, as observed in other studies [77, 78], with direct or indirect effects. Direct effects could be related to a combined effect of farming practices. First, the mechanical destruction of soil aggregates by tillage may alter microbial habitats [79]; this is supported by the higher SMMB in 2016 in link with decreased tillage and plowing. Second, fertilizer inputs can either increase SMMB following organic matter inputs or decrease it following mineral nitrogen inputs [80, 81]. Organic matter inputs were diverse (sewage sludge, hen and pig manure), but quantitatively small and much less frequent than mineral inputs. Therefore, the negative effect of farming intensity may be explained by the increase in mineral N inputs in 2016 relatively to 2011. This would also be in agreement with the increase of the SMBB in plots where P and K mineral inputs significantly decreased between 2011 and 2016. Third, the use of pesticides (herbicides and fungicides) can reduce SMMB [31]; this is consistent with the model used in this study, as the plots where SMMB significantly increased between 2011 and 2016 were those where the increase in fungicide and total pesticides was lowest. Farming intensity also had indirect negative effects on SMMB by decreasing the availability of soil resources (organic matter mineralization, dilution in the soil profile) through plowing intensity and frequency [24, 82].

Crop rotation directly modified SMMB concomitantly, negatively in 2011 but positively in 2016. This discrepancy between the 2011 and 2016 campaigns could be related to crop rotation diversity and to a greater winter crop frequency in 2016. The plots where SMMB increased between 2011 and 2016 were indeed those where the number of species in the rotation and the proportion of winter crops increased. This hypothesis is in agreement with the literature showing a positive effect of plant diversity on SMMB [83] and enhanced SMMB when the proportion of bare soils decreases thanks to winter crops [84, 85]. In addition, increasing the frequency of winter crops prevents the use of cover crops mostly composed of rapeseed and mustard in the present study and of mulching, which both negatively influence soil microbial abundance [28, 86]. The use of some plant species and their frequency in the crop rotation influence the quantity and the quality of soil resources and lead to previous-crop effects [87, 88] characterized by a significant link between crop rotations in 2011 and 2016 in the present model. Finally, the significant temporal link between SMMB in 2011 and SMMB in 2016 shows that the previous state of the microbial community directly influenced its future state under equal farming management, and its effect can sometimes be higher than treatment effects [89].

### 4.2. Soil bacterial richness

Bacterial richness was significantly structured across space and heterogeneously distributed both in 2011 and 2016, in agreement with other studies at the landscape scale [10, 90] or at larger scales [4–6, 8]. It was systematically lower in forests than in croplands, in line with the literature [69, 91]. Temporal evolutions of spatial patterns between 2011 and 2016 were not perceived at the landscape scale, but were significant when land use type was considered (i.e. croplands *vs*. forests). This is consistent with the literature: the temporal variability of microbial communities is dynamic over time, but spatial variations overcome temporal variations [92]. Our model identified drivers of soil bacterial richness and highlighted the important role of previous effects. Soil resources had a significant positive effect on bacterial richness, in accordance with other studies [18, 19]. Small variations in SOC and TN levels were observed in this study possibly leading to changes in soil bacterial richness and composition [18, 19, 93]. Rainfall and soil properties are directly linked to the dynamics of soil microbial communities [34]. Bacterial richness increased in forest soils, while water stress significantly decreased. This could be explained by an optimal functional response of bacterial richness according to the water content, as suggested in the literature [94]. Different studies mention the pH as the main driver of bacterial communities [15, 16, 18, 95]. However, its small range of variation and its lack of spatial patterns in Fénay precluded studying its effects on bacterial richness. Soil bacterial richness increases with soil texture heterogeneity [96]. As the Fénay soils were mainly clayey and silty, this would explain the negative effect of the soil habitat on soil bacterial richness.

The direct effects of farming intensity and crop rotation on bacterial richness were positive, but weaker and not very accurate in 2016. Farming practices were heterogeneous across the Fénay landscape, despite similar production situations across the whole area [97]. They changed progressively but significantly between 2011 and 2016, in agreement with observations reported at the European Union level [98]. Farmers reduced the plowing frequency, while soil bacterial richness tended to decrease or to be stable over time. This would agree with the humped back model [57]: tillage can be considered as a source of physical disturbance of the soil structure and of ecological niche components of bacterial communities [25]. A weaker disturbance led to a lower bacterial richness compared with an expected high disturbance under high plowing conditions in 2011. However, farming practices were consistent. The lower plowing frequency was associated with an increase in chemical inputs, especially herbicides since weed management is a major goal of farmers in this area. Pesticides and mineral fertilizers can negatively affect the soil bacterial diversity and structure [99], in particular ammonia-oxidizing microorganisms [100], and potentially lead to the selection or facilitation of particular bacterial taxa [31]. However, shifts in bacterial community composition do not necessarily lead to changes in bacterial richness [24]. As changes in weed management concerned the whole area, this could explain the small direct impacts of farming intensity on bacterial richness in 2016. Furthermore, an indirect negative effect of farming intensity on soil bacterial richness can occur through the modification of soil resources over short- and long-term scales depending on the frequency and intensity of farming practices [101, 102]. Soil tillage decreases TN and SOC, and thus modifies both community assemblage and composition [103].

Interestingly, crop rotation had a direct positive effect in 2011 that could be explained by a higher plant diversity in the rotation [104, 105]. Winter crop frequency increased between 2011 and 2016, and could be linked to a higher proportion of mulching in the area, which did not necessarily lead to changes in bacterial richness but had potential effects on litter properties, especially soil moisture and available phosphorus [106]. This is consistent with the direct negative effect of crop rotation on soil resources in 2011 and 2016, which was smaller in 2016. This also explains the significant indirect effects of crop rotation on bacterial richness through litter richness [107]. The crop rotation history drives soil bacterial richness through previous effects and promotes specific taxa depending on the season and crop type [87, 88]. Finally, the correlation between bacterial richness in 2011 and bacterial richness in 2016 indicated that the evolution of soil bacterial communities was dependent on their previous state [89]. This suggests that bacterial communities may respond differently according to the historical farming and land use management [108, 109].

### 4.3. Conclusion

This study shows that the evolution of soil microbial communities depends on both soil properties and farming practices. Previous-crop effects and the historical farming management were identified as determining factors for a better understanding of soil microbial variations at the landscape scale. Taking past farming management into account is important to understand the temporal evolution of soil microbial communities: a same farming practice may not have the same impact on two different farming plots due to previous-crop and management effects. However, some practices like a lower plowing frequency, a more diverse crop rotation should benefit microbial communities, whereas chemical inputs benefit to only a few microbial species and decrease richness. To go further, the bacterial community composition needs to be described in order to understand how each taxon is impacted by farming practices. This study is a first step toward creating a predictive model to evaluate soil microbial properties depending on farming management. The PLS PM method is a suitable method for identifying important mechanisms for our predictive model.

## Supporting information

S1 Table Direct, indirect and total effects of LVs in the PLS-PM models for soil molecular microbial biomass and bacterial richness. Only non-zero effects are shown.
(DOCX)

S1 File Terrat, comparison between the pyrosequencing and Illumina sequencing technologies. (DOCX)
