# Correction: Soil microbial communities in the face of changing farming practices: A case study in an agricultural landscape in France

**DOI:** 10.1371/journal.pone.0350312

**Published:** 2026-05-28

**Authors:** Laurie Dunn, Christophe Lang, Nicolas Marilleau, Sébastien Terrat, Luc Biju-Duval, Mélanie Lelièvre, Solène Perrin, Nicolas Chemidlin Prévost-Bouré

In Fig 3 of [1], the first map representing organic carbon over the landscape in 2011 appears blank. Please see the correct Fig 3 here.

In Fig 6 of [1], a scatter plot for SMMB has been included in error. The scatter plot should be for soil bacterial richness. Please see the correct Fig 6 here.

**Fig 3:**
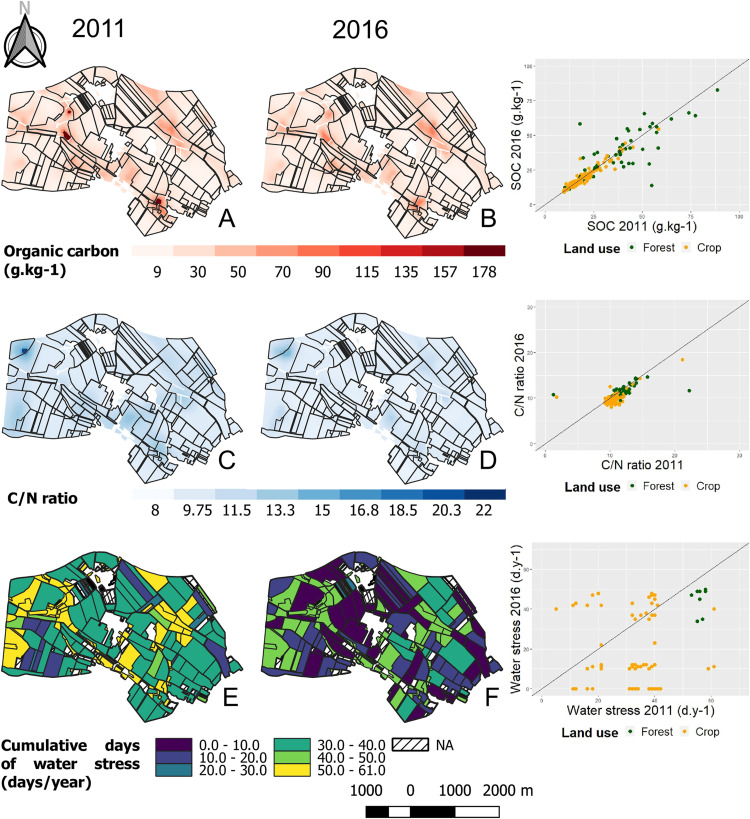
Interpolated mapping of the soil variables organic carbon(A, B), C:N ratio (C, D) and cumulative days of water stress (E, F) for the 2011 sampling campaign (A, C, E) and the 2016 campaign (B, D, F).The Matérn model was used to fit the experimental variogram. The ranges of the models were A: 144.33 m; B: 126.70 m; C: 126.24 m; D: 114.63 m. The estimated water stress in the topsoil is represented at the plot scale. Scatter plots on the right of the figure correspond to the variation of the parameter in 2016 depending on the same parameter in 2011. Green dots, forest plots; orange dots, farmed plots. Plot lines, equation y = x.

**Fig 6:**
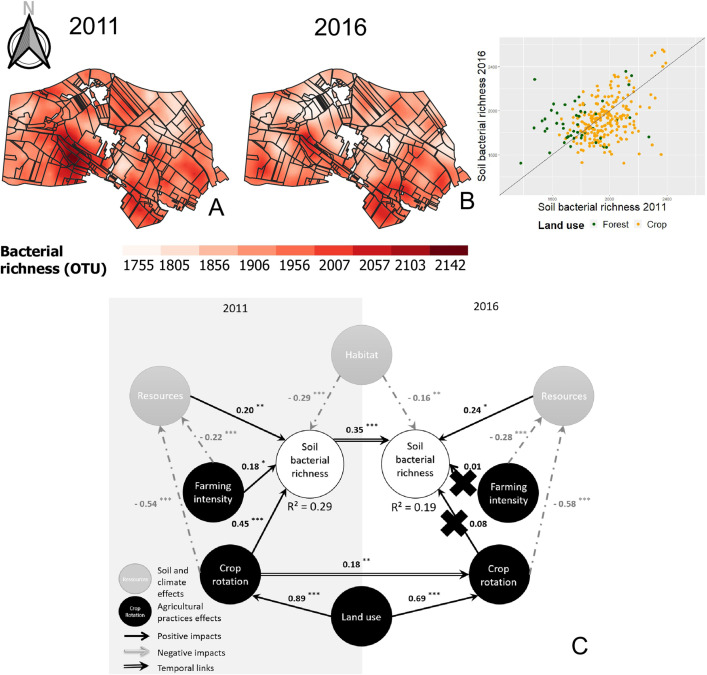
Interpolated mapping of soil bacterial richness in 2011 (A) and 2016(B), and complete path model (C). In the interpolated maps, the color scale is the same for the two sampling campaigns and indicates the extrapolated values. The ranges of the models were 349.00 m (A) and 101.35 m (B). The scatter plot on the right of the maps corresponds to the variation of the parameter in 2016 depending on the same parameter in 2011. Green dots, forest plots; orange dots, farmed plots. Line, equation y =x. Concerning the complete path model for biomass analysis (C), circles correspond to the latent variables (LVs), with anthropogenic LVs in black and ecological LVs in gray. Path coefficients were computed from regressions and allowed us to estimate the strength and direction of relations between LVs. Black arrow, positive impact; gray arrow, negative impact; double arrow, temporal impact. Significant impacts were evaluated based on a t-test: *P* < 0.1; *: *P* < 0.05; **: *P* < 0.01; ***: *P* < 0.001). Crosses indicate inaccurate path coefficients according to a bootstrap validation.
