# Supplementary material for: Soil microbial communities in the face of changing farming practices: A case study in an agricultural landscape in France
Source: PLoS One. 2021 Jun 17;16(6):e0252216. doi: 10.1371/journal.pone.0252216 (PMC8211295; doi:10.1371/journal.pone.0252216)
Supplement: S1 Table — Only non-zero effects are shown. (DOCX) [file pone.0252216.s001.docx]

**S1 Table. Direct, indirect and total effects of LVs in the PLS-PM models for soil molecular microbial biomass and bacterial richness.** Only non-zero effects are shown.

|  | Soil molecular microbial biomass analysis |  |  | direct | indirect | total |
| --- | --- | --- | --- | --- | --- | --- |
| Soil characteristics | Habitat | -> | Soil microbial biomass 2011 | 0.13 | 0.00 | 0.13 |
|  | Habitat | -> | Soil microbial biomass 2016 | 0.01 | 0.03 | 0.04 |
|  | Resources 2011 | -> | Soil microbial biomass 2011 | 0.61 | 0.00 | 0.61 |
|  | Resources 2011 | -> | Soil microbial biomass 2016 | 0.00 | 0.14 | 0.14 |
|  | Resources 2016 | -> | Soil microbial biomass 2016 | 0.62 | 0.00 | 0.62 |
| Farming practices | LUCrop | -> | Crop Rotation 2011 | 0.89 | 0.00 | 0.89 |
|  | LUCrop | -> | Resources 2011 | 0.00 | -0.47 | -0.47 |
|  | LUCrop | -> | Soil microbial biomass 2011 | 0.00 | -0.38 | -0.38 |
|  | LUCrop | -> | Crop Rotation 2016 | 0.70 | 0.16 | 0.86 |
|  | LUCrop | -> | Resources 2016 | 0.00 | -0.46 | -0.46 |
|  | LUCrop | -> | Soil microbial biomass 2016 | 0.00 | -0.25 | -0.25 |
|  | Crop Rotation 2011 | -> | Resources 2011 | -0.53 | 0.00 | -0.52 |
|  | Crop Rotation 2011 | -> | Soil microbial biomass 2011 | -0.11 | -0.32 | -0.43 |
|  | Crop Rotation 2011 | -> | Resources 2016 | 0.00 | -0.10 | -0.10 |
|  | Farming intensity 2011 | -> | Resources 2011 | -0.22 | 0.00 | -0.22 |
|  | Farming intensity 2011 | -> | Soil microbial biomass 2011 | -0.16 | -0.13 | -0.30 |
|  | Crop Rotation 2016 | -> | Resources 2016 | -0.54 | 0.00 | -0.54 |
|  | Crop Rotation 2016 | -> | Soil microbial biomass 2016 | 0.15 | -0.34 | -0.18 |
|  | Farming intensity 2016 | -> | Resources 2016 | -0.27 | 0.00 | -0.27 |
|  | Farming intensity 2016 | -> | Soil microbial biomass 2016 | -0.14 | -0.17 | -0.31 |
|  | Crop Rotation 2011 | -> | Crop Rotation 2016 | 0.18 | 0.00 | 0.18 |
| Previous effects | Crop Rotation 2011 | -> | Soil microbial biomass 2016 | 0.00 | -0.13 | -0.13 |
|  | Farming intensity 2011 | -> | Soil microbial biomass 2016 | 0.00 | -0.07 | -0.07 |
|  | Soil microbial biomass 2011 | -> | Soil microbial biomass 2016 | 0.23 | 0.00 | 0.23 |
|  | Richness analysis |  |  | direct | indirect | total |
| Soil characteristics | Habitat | -> | Soil bacterial richness 2011 | -0.29 | 0.00 | -0.29 |
|  | Habitat | -> | Soil bacterial richness 2016 | -0.17 | -0.10 | -0.27 |
|  | Resources 2011 | -> | Soil bacterial richness 2011 | 0.20 | 0.00 | 0.20 |
|  | Resources 2011 | -> | Soil bacterial richness 2016 | 0.00 | 0.07 | 0.07 |
|  | Resources 2016 | -> | Soil bacterial richness 2016 | 0.24 | 0.00 | 0.24 |
| Farming practices | LUCrop | -> | Crop Rotation 2011 | 0.89 | 0.00 | 0.89 |
|  | LUCrop | -> | Resources 2011 | 0.00 | -0.48 | -0.48 |
|  | LUCrop | -> | Soil bacterial richness 2011 | 0.00 | 0.30 | 0.30 |
|  | LUCrop | -> | Crop Rotation 2016 | 0.69 | 0.16 | 0.85 |
|  | LUCrop | -> | Resources 2016 | 0.00 | -0.49 | -0.49 |
|  | LUCrop | -> | Soil bacterial richness 2016 | 0.00 | 0.06 | 0.06 |
|  | Crop Rotation 2011 | -> | Resources 2011 | -0.54 | 0.00 | -0.54 |
|  | Crop Rotation 2011 | -> | Soil bacterial richness 2011 | 0.44 | -0.11 | 0.34 |
|  | Crop Rotation 2011 | -> | Crop Rotation 2016 | 0.18 | 0.00 | 0.18 |
|  | Crop Rotation 2011 | -> | Resources 2016 | 0.00 | -0.11 | -0.11 |
|  | Farming intensity 2011 | -> | Resources 2011 | -0.22 | 0.00 | -0.22 |
|  | Farming intensity 2011 | -> | Soil bacterial richness 2011 | 0.18 | -0.05 | 0.14 |
|  | Crop Rotation 2016 | -> | Resources 2016 | -0.58 | 0.00 | -0.58 |
|  | Crop Rotation 2016 | -> | Soil bacterial richness 2016 | 0.08 | -0.14 | -0.05 |
|  | Farming intensity 2016 | -> | Resources 2016 | -0.28 | 0.00 | -0.28 |
|  | Farming intensity 2016 | -> | Soil bacterial richness 2016 | 0.01 | -0.07 | -0.06 |
| **Previous effects** | Crop Rotation 2011 | -> | Soil bacterial richness 2016 | 0.00 | 0.11 | 0.11 |
|  | Farming intensity 2011 | -> | Soil bacterial richness 2016 | 0.00 | 0.05 | 0.05 |
|  | Soil bacterial richness 2011 | -> | Soil bacterial richness 2016 | 0.35 | 0.00 | 0.35 |
