# Supplementary material for: Soil microbial communities in the face of changing farming practices: A case study in an agricultural landscape in France
Source: PLoS One. 2021 Jun 17;16(6):e0252216. doi: 10.1371/journal.pone.0252216 (PMC8211295; doi:10.1371/journal.pone.0252216)
Supplement: S1 File — (DOCX) [file pone.0252216.s002.docx]

**S1 File: Comparison of 454 (Roche) and Illumina sequencing results dedicated to the characterization of soil microbial communities**

**Introduction**

Determining the soil microbial community structure by means of amplicon sequencing (using next-generation sequencing – NGS) is now a well-known approach. However, whatever the NGS approach, several crucial steps can introduce biases and therefore affect the final results, such as DNA extraction and isolation [1], primer definition and the PCR procedure [2], the sequencing technology [3, 4], data analysis [5]. Taking all these biases into account is a crucial step of the analysis when comparing datasets from large temporal of spatial scales.

The main current problem in link with these steps is the fast evolution of sequencing technologies. The Roche 454, Illumina/Solexa technologies, and ABI SOLiD to a lesser extent, have been extensively used to characterize soil microbial communities. However, these technologies are either constantly upgraded, requiring regular optimizations and verifications, or dropped down. Thus, in 2013 Roche announced that they would stop the production of 454 sequencers (released in 2005) and support by 2016.

Assessing sequence quality is critical. To this end, several studies have attempted to evaluate the sequencing errors and artifacts specific to each NGS platform [4]. Yet, we compared the Roche 454 FLX Titanium and Illumina MiSeq technologies again by evaluating the alpha diversity estimates (including number of OTUs, Chao1, ACE, Shannon Wiener’s and Simpson’s evenness estimates) of several soil samples to provide new insights into the direct comparison of these two technologies.

**Materials & methods**

Sampling design

Soil samples were obtained from the French Soil Quality Monitoring Network (“Réseau de Mesures de la Qualité des Sols”, RMQS), a soil monitoring network based on a 16-km regular grid across the 550,000 km^2^ of the French territory [6, 7]. All samples were collected between 2002 and 2009. All sites were geo-positioned with <0.5 m precision, and 25 individual core samples were taken from the topsoil (0–30 cm) in the middle of each 16 × 16 km square using an unaligned sampling design within a 20 × 20 m area. These core samples were bulked to obtain a composite sample for each RMQS site. The soil samples were gently air-dried, sieved to 2 mm and then stored at −40 °C before analysis. Thirty soil samples were randomly selected from the global collection of 2,173 RMQS samples.

Molecular characterization of microbial communities

To evaluate the sole impact of the sequencing technologies, the analysis of these 30 samples required the rigorous standardization of the range of different molecular tools involved in soil DNA extraction and amplification. The protocols described below were applied.

*Soil DNA extraction and purification*

Microbial DNA was extracted and purified from 1 g of each of the 30 composite soils sampled at each RMQS site, using the previously described GnS-GII procedure [1]. Crude DNA extracts were quantified by agarose gel electrophoresis stained with ethidium bromide, using calf thymus DNA to draw a standard curve [6]. Crude DNA was then purified using a MinElute gel extraction kit (Qiagen, France) and quantified using a QuantiFluor staining kit (Promega, USA), prior to further investigations.

*PCR amplification and sequencing of 16S rRNA gene sequences*

A 16S rRNA gene fragment targeting the V3-V4 regions was amplified to characterize bacterial diversity using the primers F479 (5′-CAGCMGCYGCNGTAANAC-3′) and R888 (5′-CCGYCAATTCMTTTRAGT-3′) following the method previously described in [1]. The PCR products were then purified using a MinElute PCR purification kit (Qiagen) and quantified using a QuantiFluor staining kit (Promega, USA). A second PCR of 7 cycles was then run twice for each sample under similar PCR conditions, with purified PCR products as matrices (7.5 ng of DNA were used for a 25 µL PCR mix), and dedicated fusion primers (‘MID/F479, ‘MID/R888) with the multiplex identifiers at the 5′ ends. All duplicated PCR products were then pooled, purified using a MinElute PCR purification kit (Qiagen), and quantified using a QuantiFluor staining kit (Promega, USA). Equal amounts from the 30 samples were pooled, and then cleaned to remove excess nucleotides, salts and enzymes using the Agencourt AMPure XP system (Beckman Coulter Genomics). One hundred microliters of TE buffer (Roche) were used for the elution. The library was then treated for Roche 454 FLX Titanium sequencing or Illumina MiSeq sequencing, adding dedicated adaptors. The two sequencing approaches were carried out by the Genoscope (Evry, France, https://www.cea.fr/drf/ifrancoisjacob/Pages/Departements/Genoscope.aspx).

*Bioinformatics sequence analysis*

Bioinformatic analyses were done using the BIOCOM-PIPE pipeline, with the same parameters as those previously described in [7, 8] and in the main manuscript. After sequencing, 637,456 raw reads were obtained for the 30 soil samples using Roche 454 FLX Titanium, and 2,170,472 raw reads using Illumina MiSeq. After the different preprocessing and filtering steps (detailed in the main manuscript), the number of high-quality reads for each sample was rarefied (i.e. 10,000 high-quality reads for each sample) by random selection to allow for an efficient comparison of the datasets and avoid biased community comparisons and rarefaction curves. Thus, only 23 soil samples out of the 30 initial samples were kept for subsequent analyses, encompassing a total of 46,000 reads. Finally, all retained reads were aligned with INFERNAL [9] and clustered into OTUs at 95% similarity against the database of known reads from the complete RMQS dataset, using ReClustOR [8]. All raw datasets are publicly available in the EBI database system (in the Short Read Archive) under project accession no XXX.

**Results & Discussion**

The ReClustOR algorithm set with a cutoff at 95% sequence identity was applied to the 23 samples sequenced with both technologies issued 36,192 OTUs. The Roche 454 technology showed 2,293 OTUs on average (minimum 1,502; maximum 2,968) and Illumina 2,163 on average (minimum 1,360; maximum 2,848). The comparison of the two datasets highlighted a high correlation besides small differences in terms of OTUs (see S1 Fig). The high r^2^ (0.968) of the linear regression revealed that general trends in terms of OTUs were conserved and matched well regardless of the sequencing technology. Taking a closer look at the number of OTUs, richness was systematically underestimated by Illumina (around 5.94% of the average value; minimum 0.60%, maximum 11.97%). This kind of result was highlighted recently [3], and one hypothesis is an effect of the additional PCR step in the preparation of the library. The higher number of OTUs can also be a consequence of the treatment of homopolymer differences, leading to a slight loss of OTUs by the Illumina technology.


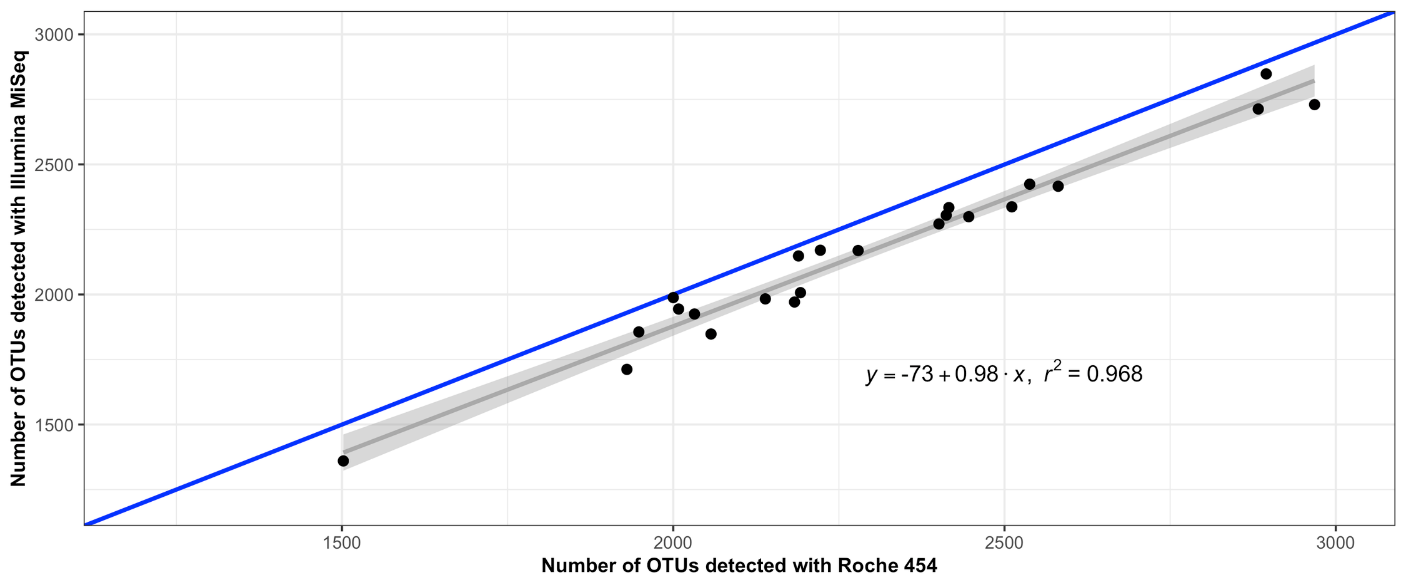


**S1 Fig: Comparison of the Roche 454 and Illumina MiSeq technologies results in terms of detected OTUs.** Blue line, unity line. A regression line (in gray) was also computed between the two datasets.

**Conclusion**

Comparing Roche 454 GS FLX Titanium and Illumina MiSeq by using the same PCR primers and bioinformatic analyses resulted in corresponding trends in richness. Therefore, datasets issued by different sequencing technologies can be grouped together.

**References**

1. Terrat S, Plassart P, Bourgeois E, Ferreira S, Dequiedt S, Adele-Dit-De-Renseville N, et al. Meta-barcoded evaluation of the ISO standard 11063 DNA extraction procedure to characterize soil bacterial and fungal community diversity and composition. Microb Biotechnol 2015; 8: 131–142.

2. Tremblay J, Singh K, Fern A, Kirton ES, He S, Woyke T, et al. Primer and platform effects on 16S rRNA tag sequencing. Front Microbiol 2015; 6: 1–15.

3. Sinclair L, Osman OA, Bertilsson S, Eiler A. Microbial community composition and diversity via 16S rRNA gene amplicons: Evaluating the illumina platform. PLoS One 2015; 10: 1–18.

4. Luo C, Tsementzi D, Kyrpides N, Read T, Konstantinidis KT. Direct comparisons of Illumina vs. Roche 454 sequencing technologies on the same microbial community DNA sample. PLoS One 2012; 7.

5. Escudié F, Auer L, Bernard M, Mariadassou M, Cauquil L, Vidal K, et al. FROGS: Find, Rapidly, OTUs with Galaxy Solution. Bioinformatics 2017; 1–8.

6. Dequiedt S, Thioulouse J, Jolivet C, Saby NPA, Lelievre M, Maron P-A, et al. Biogeographical patterns of soil bacterial communities. Environ Microbiol Rep 2009; 1: 251–255.

7. Terrat S, Horrigue W, Dequiedt S, A Saby NP, Lelièvre M, Nowak V, et al. Correction: Mapping and predictive variations of soil bacterial richness across France. PLoS One 2017; 12: e0186766.

8. Terrat S, Djemiel C, Journay C, Karimi B, Dequiedt S, Horrigue W, et al. ReClustOR : a re‐clustering tool using an open‐reference method that improves operational taxonomic unit definition. Methods Ecol Evol 2020; 2020: 168–180.

9. Nawrocki EP, Eddy SR. Infernal 1.1: 100-fold faster RNA homology searches. Bioinformatics 2013; 29: 2933–2935.
